# NK1-r Antagonist Treatment Comparable to Decompressive Craniectomy in Reducing Intracranial Pressure Following Stroke

**DOI:** 10.3389/fnins.2019.00681

**Published:** 2019-07-05

**Authors:** Annabel J. Sorby-Adams, Anna V. Leonard, Jan W. Hoving, Nawaf Yassi, Robert Vink, Adam J. Wells, Renée J. Turner

**Affiliations:** ^1^Adelaide Medical School and Adelaide Centre for Neuroscience Research, The University of Adelaide, Adelaide, SA, Australia; ^2^Department of Medicine and Neurology, Melbourne Brain Centre at the Royal Melbourne Hospital, The University of Melbourne, Parkville, VIC, Australia; ^3^Department of Radiology, Amsterdam UMC, University of Amsterdam, Amsterdam, Netherlands; ^4^Florey Institute of Neuroscience and Mental Health, Parkville, VIC, Australia; ^5^Division of Health Sciences, University of South Australia, Adelaide, SA, Australia; ^6^Department of Neurosurgery, Royal Adelaide Hospital, Adelaide, SA, Australia

**Keywords:** intracranial pressure, cerebral edema, substance P, decompressive craniectomy, blood-brain barrier, stroke

## Abstract

**Background and Purpose:** The morbidity and early mortality associated with stroke is largely attributable to cerebral edema and elevated intracranial pressure (ICP). Existing pharmacotherapies do not target the underlying pathophysiology and are often ineffective in sustainably lowering ICP, whilst decompressive craniectomy (DC) surgery is life-saving yet with surgical/peri-operative risk and increased morbidity in the elderly. Accordingly, there is an urgent need for therapies that directly target the mechanisms of edema genesis. Neurogenic inflammation, mediated by substance P (SP) binding to the tachykinin NK1 receptor (NK1-r), is associated with blood-brain barrier (BBB) disruption, cerebral edema and poor outcome post-stroke. NK1-r antagonist treatment ameliorates BBB dysfunction and cerebral edema in rodent stroke models. However, treatment has not been investigated in a large animal model, an important step toward clinical translation. Consequently, the current study compared the efficacy of NK1-r antagonist treatment to DC surgery in reducing ICP post-stroke in a clinically relevant ovine model.

**Methods:** Anesthetized female Merino sheep (65 ± 6 kg, 18–24 months) underwent sham surgery (*n* = 4) or permanent middle cerebral artery occlusion (*n* = 22). Stroke animals were randomized into one of 5 treatments: 1×NK1 bolus (4 h), 2×NK1 bolus (4 h;9 h), 3×NK1 bolus (4 h;9 h;14 h), DC surgery (performed at 4 h) or saline vehicle. ICP, blood pressure and blood gasses were monitored for 24 h post-stroke. At 24 h post-stroke anesthetized animals underwent MRI followed by perfusion and brains removed and processed for histological assessment.

**Results:** 2×NK1, 3×NK1 administration or DC surgery significantly (*p* < 0.05) reduced ICP compared to vehicle. 1×NK1 was ineffective in sustainably lowering ICP. On MRI, midline shift and cerebral edema were more marked in vehicles compared to NK1-r treatment groups.

**Conclusion:** Two or three boluses of NK1-r antagonist treatment reduced ICP comparable to DC surgery, suggesting it may provide a novel alternative to invasive surgery for the management of elevated ICP.

## Introduction

Elevated intracranial pressure (ICP) arising as a result of malignant cerebral edema is the leading cause of death in the first week following stroke (Hacke et al., 1996). Despite the devastating impact of elevated ICP on patient outcome, clinical management remains sub-optimal. Pharmacological interventions for ICP management are limited, and while osmotic therapies (including mannitol and hypertonic saline) are used in some centers (Zhang et al., 2018), there is no evidence that these therapies are independently effective in improving outcome. Furthermore, the efficacy of other pharmacotherapies including corticosteroids and barbiturates remains ambiguous for the treatment of post-stroke cerebral edema (Brogan and Manno, 2015). In selected patients with malignant middle cerebral artery (MCA) territory infarction, surgical intervention with decompressive craniectomy (DC) may be required. This procedure rapidly alleviates pressure by removing a large portion of the skull and opening the dura overlying the brain, thereby providing space for the edematous brain to swell freely until cerebral edema resolves, typically beyond the first week following stroke (Xiao-feng et al., 2005). However, although this procedure reduces compression on cerebral structures and the risk of life-threatening tonsillar herniation, it is also associated with increased morbidity in those aged greater than 60 years, the patient population in which stroke is most prevalent (Jüttler et al., 2007; Das et al., 2019). It is clear that current pharmacological and surgical interventions are inadequate as they target the symptoms, rather than the underlying cause of cerebral edema and concomitant rise in ICP (Bardutzky and Schwab, 2007; Simard et al., 2007). An enhanced understanding of the mechanisms that underlie the genesis of cerebral edema and raised ICP is critical for the development of more targeted and effective treatments.

Although factors associated with the pathogenesis of cerebral edema are not completely understood, neurogenic inflammation has been identified as a potential therapeutic target (Stumm et al., 2001; Turner et al., 2006; Corrigan et al., 2016a). Neurogenic inflammation is a neurally mediated process involving the release of neuropeptides, including substance P (SP) and calcitonin gene-related peptide, which initiate vasodilation, increased microvascular permeability and edema (Hokfelt et al., 2000). Although well established as a precipitant of edema development within peripheral tissues such as the skin and lungs (Alves et al., 1999), neurogenic inflammation remained largely unexplored as a potential mechanism of cerebral edema development (Lewis et al., 2013; Sorby-Adams et al., 2017). This was until studies depleting neuropeptides or blocking the action of SP at the tachykinin NK1 receptor (NK1-r), to which SP binds preferentially, showed a ubiquitous role for this process in the genesis of cerebral edema following stroke and traumatic brain injury (TBI) (Vink et al., 2003; Nimmo et al., 2004; Turner et al., 2006, 2011; Donkin et al., 2009; Turner and Vink, 2012, 2014; Corrigan et al., 2016a). Specifically, increased SP immunoreactivity within the penumbral perivascular tissue is associated with profound BBB disruption and cerebral edema at 24 h post-stroke and poor functional outcomes up to 7 days following middle cerebral artery occlusion (MCAO) in rats, with NK1-r blockade ameliorating these effects (Turner et al., 2011; Turner and Vink, 2012, 2014; Corrigan et al., 2016a). Increased plasma SP levels have also been reported clinically following severe acute ischemic stroke, elevated levels of which were associated with increased mortality (Lorente et al., 2016). Comparable alterations in SP levels have also been observed in clinical TBI correlating with injury severity and mortality (Lorente et al., 2015).

The cumulative evidence from these studies suggests an important role for SP in the development of cerebral edema and poor outcome following acute CNS injury. However, pre-clinical stroke evaluation of the NK1-r antagonist has been limited to small animal stroke models, with the most extensive screening to date performed in small and large animal models of TBI. Whilst rodent species are essential for establishing basic biological processes, it is often difficult to obtain clinically relevant outcome measures, thereby limiting clinical translation. Given the extremely poor translation of stroke neuroprotection from the laboratory to the clinic (Xu and Pan, 2013), evaluation of promising therapies in a large, intermediate species is essential. Species such as sheep are an ideal candidate for such studies due to the ability to utilize clinically relevant outcome measures in addition to similarities in neuroanatomical structure, including gyrencephalic cerebral organization and significant portion of white matter (Sorby-Adams et al., 2018). Of particular relevance when investigating elevated ICP, however, is the strong tentorium cerebelli of the sheep, separating supra- and infratentorial compartments, bearing structural similarity to that of the human (Dostovic et al., 2016). In the setting of clinical malignant cerebral edema, this leads to compartmentalization of pressure within the supratentorial space, tonsillar herniation, and ultimately often fatal compression of the brain stem as the brain attempts to alleviate dangerously elevated ICP. Indeed, ovine stroke models replicate key clinical hallmarks following stroke including elevated ICP, cerebral edema, midline shift and tonsillar herniation (Wells et al., 2012; Wells et al., 2015; Sorby-Adams et al., 2018).

As such, the aim of the present study was to determine the effect of inhibition of SP with an NK1-r antagonist, compared to DC surgery, on ICP following permanent ischemic stroke in an ovine model.

## Materials and Methods

### Animals and Experimental Design

Female Merino sheep (***n*** = 26) aged 18–24 months old (65 ± 6 kg) were allocated to the study. Animals were randomized into either sham surgery or permanent MCA occlusion (MCAO). Following induction of stroke animals were then randomized into one of the following groups: (1) saline vehicle treatment at 4 h post-stroke (***n*** = 6); (2) 1**×** bolus NK1 antagonist at 4 h post-stroke (***n*** = 3); (3) 2**×** boluses of NK1 antagonist at 4 h and 9 h post-stroke (***n*** = 3); (4) 3**×** boluses of NK1 antagonist at 4, 9 and 14 h post-stroke (***n*** = 6); or (5) decompressive craniectomy (***n*** = 5) at 4 h post-stroke. Note that the dose for the NK1-r antagonist treatment was the same across all the NK1-r treatment groups, the only difference was the number of boluses that each group received, either one (1**×**NK1), two (2**×**NK1), or three (3**×**NK1) boluses. The NK1-r antagonist used in the study was a test compound supplied by PresSuraNeuro prepared at a concentration of 1 mg/ml in warmed saline (37°C) and administered at a dose of 1 ml/kg via an indwelling jugular venous catheter, as per previous studies in our laboratory (Vink et al., 2017).

### Anesthesia and Physiological Monitoring

Anesthesia was induced with intravenous thiopentone (1000 mg in 20 mL, Jurox Pty Ltd., Australia) and maintained with 1.5% inhalational isoflurane (Veterinary Companies of Australia Pty Ltd., Australia) in a mixture of oxygen and room air (500:5000 ml/min), plus intravenous ketamine (Parnell Australia Pty Ltd., Australia) infusion at 4 mg/kg/hr via a femoral venous line. These two anesthetic agents were used in combination to avoid the intrinsic neuroprotective properties of either above certain doses, and to maintain a twilight general anesthesia (Hudetz and Pagel, 2010; Schifilliti et al., 2010). With the animal supine, an arterial catheter was placed in the right femoral artery for continuous blood pressure monitoring and periodic arterial blood gas sampling, and a venous catheter inserted for anesthetic and fluid administration. The animal was then placed prone in the sphinx position, a burr hole drilled in the right parietal bone posterior to the coronal suture and approximately 20 mm from the sagittal suture, dura perforated, and skull bolts secured. A Codman microsensor ICP probe (Codman & Shurtleff Inc., MA, United States) was introduced into the bolt, calibrated and inserted intraparenchymally to measure ICP within the supratentorial compartment. Using LabChart Reader (v 8.1.1), mean arterial blood pressure (MABP) and ICP were continuously recorded through the 24 h monitoring period. Arterial blood gas sampling was conducted prior to MCAO or sham surgery and at hourly intervals throughout the monitoring period until the completion of the experiment to maintain physiological pO_2_, pCO_2_, and pH.

### Surgical Approach to MCAO

We have previously described the surgical approach to proximal MCAO in detail (Wells et al., 2012, 2015). Briefly, a 50 mm vertical incision was made posterior to the right eye, terminating at the zygomatic arch. The underlying muscle was retracted, coronoid process removed, and the skull revealed. A small craniotomy was performed at the junction of the parietal and squamous temporal bones with a high-speed pneumatic drill using a 5 mm cutting burr (Midas Rex, Medtronic, MN, United States). All intra-dural work was carried out with loupe magnification and a head mounted light source (Surgical Acuity, WI, United States). A horse-shoe shaped durotomy was performed with an inferiorly based flap allowing for gentle upward retraction of the anterior temporal lobe to achieve visualization of the proximal MCA. Animals then either underwent sham surgery in which the proximal MCA was dissected but not occluded, or permanent MCA in which the proximal MCA was occluded via Malis bipolar diathermy forceps (Valleylab Inc., CO, United States). The exposed brain was irrigated with saline during surgery to prevent dehydration of the cerebral cortex. After completion of sham or MCAO surgery the dura was approximated and synthetic dura (Durepair, Medtronic) interleaved under the existing dura (in the case of dural retraction following incision) and closed watertight with ethyl cyanoacrylate (Bostik, Australia). Once dural closure was confirmed and no leakage of CSF observed, the craniotomy site was reinforced with dental acrylic cement (Lang Dental, IL, United States) that was manipulated into the edge of the craniotomy and the wound closed in layers, maintaining the shape of the cranial cavity and importantly, the homeostasis of ICP dynamics. For animals allocated to the DC group, at 4h following stroke the wound was opened, dental acrylic removed, and the craniotomy site widened (3 cm **×** 6 cm) to allow for decompression. A dural pouch was created using Durepair to expand the intracranial cavity, after which the bone was left off and the overlying skin sutured closed, simulating clinical DC and expansile duraplasty. For all groups, the head was then returned to a neutral position for monitoring under general anesthesia.

### Magnetic Resonance Imaging

At 24 h following stroke or sham surgery onset animals were placed under general anesthesia (3% isoflurane) in a 1.5 Tesla Siemens Sonata (Siemens AG, Munich, Germany) magnetic resonance imaging (MRI) scanner. The scanning sequence included time-of-flight magnetic resonance angiography (TOF MRA; TR 26.0 ms, TE 3.69 ms, slice thickness 0.50 mm, slices per slab 48), diffusion weighted imaging (DWI; TR 5600.0 ms, TE 80 ms, slice thickness 3.0 mm, slices per slab 25), fluid attenuated inversion recovery (FLAIR; TR 5000.0 ms, TE 386 ms, slice thickness 0.9 mm, slices per slab 96), T1 (TR 2300.0 ms, TE 2.58 ms, slice thickness 0.9 mm, slices per slab 96) and T2 (TR 3200 ms, TE 410 ms, slice thickness 0.9 mm, slices per slab 96) weighted images.

The degree of midline shift on MRI was used as a marker of the amount of expansion of the infarcted hemisphere, indicative of cerebral edema. Extent of shift from the midline was assessed using axial T2-weighted scans and measured in mm from the septum pellucidum at the level of the foramen of Monro (Horos DICOM image viewer v3.1.1). To calculate cerebral edema, coronal FLAIR images were analyzed using Horos (v3.1.1). After optimal adjustment of brightness and contrast, edema volume was determined from sequences using computer-aided manual tracing of the hyperintense lesion by a blinded assessor. The areas were then summed and multiplied by the slice thickness to give a total volume in cm^3^.

To calculate infarct volume, segmentation tools in ITK-SNAP (v 3.7) were used to perform semi-automated segmentation of the MRI diffusion lesions using diffusion-weighted images (Yushkevich et al., 2006). A combination of “three-dimensional active contour segmentation” and subsequent manual post-processing of the segmentation while adjusting image thresholds was performed to maximize reproducibility whilst excluding artifacts. The “three-dimensional active contour segmentation” consisted of multiple steps: First, in the pre-segmentation phase, independent component analysis automatically segmented parts of the DWI image and these were manually identified as foreground or background. To obtain an optimal distinction between foreground and background, thresholds of the image windows were adjusted. After thresholding, a “speed image” with a separate foreground and background was created. Next, in the active contour phase, seed regions were manually placed within the region of interest (ROI). These seeds were then automatically grown within the ROIs to form the temporary segmentation. Subsequently, areas that were not automatically included in the “active contour segmentation,” were manually included in the follow-up infarct segmentation. The total volume of the segmentation was exported and reported in cm^3^ for analysis.

### Histological Examination

Following MRI, intravenous heparin (5000I.U./5 ml; Pfizer, NY, United States) was administered and animals euthanized via common carotid perfusion fixation with cold Tris–buffered saline under Isoflurane anesthesia. The brains were subsequently removed and sliced into 10 mm coronal slices using a custom-made matrix. Sections were then immersion fixed in 10% neutral-buffered formalin for a minimum of 14 days prior to being processed, embedded in paraffin wax and sectioned coronally at 5 micron intervals for histological examination by hematoxylin and eosin (H&E), albumin (1:2000, Dako Pty Ltd., A0001), SP (1:5000 citrate retrieval, Abcam Pty Ltd., ab14184), NKI-r (1:1000 citrate retrieval, Advanced Targeting Systems Pty Ltd., AB-N-33AP) and caveolin-1 (cav-1; 1:1000 EDTA retrieval, Cell Signaling Technologies Pty Ltd., 3238) immunohistochemistry.

### Statistical Analysis

All data are expressed as mean ± SD. Physiological data (arterial blood pressure, pH, pO_2_, pCO_2_) were analyzed using one-way analysis of variance (ANOVA) followed by Tukey’s *post hoc* tests (Prism v.8.0.1, Graphpad, CA, United States). Values were averaged for each treatment group across all time points and reported as a single value. Raw ICP and MABP data underwent a logarithmic exponential transformation as previously described and were expressed as geometric mean ± SD (Matthews et al., 1990; Wells et al., 2015). ICP was analyzed by two-way ANOVA and lesion volume, cerebral edema and midline shift data were analyzed by one-way ANOVA, all followed by Tukey’s *post hoc* tests. A *p*-value of < 0.05 was considered significant. Correlations were performed between cerebral edema and midline shift and ICP and cerebral edema to determine the relationship between these variables.

## Results

### Surgery, Mortality and Physiological Parameters

All experimental procedures were carried out without complication and there was no premature mortality or unexpected events in any of the groups. Basic physiological parameters are expressed in Table 1. For all groups, there was no statistically significant difference in pO_2_, pCO_2_, or pH at any of the time-points following stroke. However, there was a statistically significant difference in MABP between sham, 1×NK1 (*p* = 0.02) DC (*p* = 0.02) and 3×NK1 (*p* = 0.006) treatment groups.

**Table 1:** Basic physiological parameters.

Group	pCO_2_ (mmHg) ± SD	pO_2_ (mmHg) ± SD	MABP (mmHg) ± SD	pH (-log [H^+^]) ± SD
Sham	43 ± 8.41	113 ± 14.09	79 ± 8.78	7.48 ± 0.039
Vehicle	40 ± 6.09	127 ± 31.28	104 ± 10.17	7.46 ± 0.062
1×NK1	36 ± 2.55	138 ± 25.06	112 ± 10.18	7.51 ± 0.033
2×NK1	43 ± 6.56	149 ± 33.99	98 ± 5.81	7.45 ± 0.031
3×NK1	37 ± 2.34	135 ± 20.53	111 ± 15.66	7.52 ± 0.018
DC	42 ± 3.78	150 ± 17.22	108 ± 10.65	7.45 ± 0.082

### Intracranial Pressure

**Figure 1:**
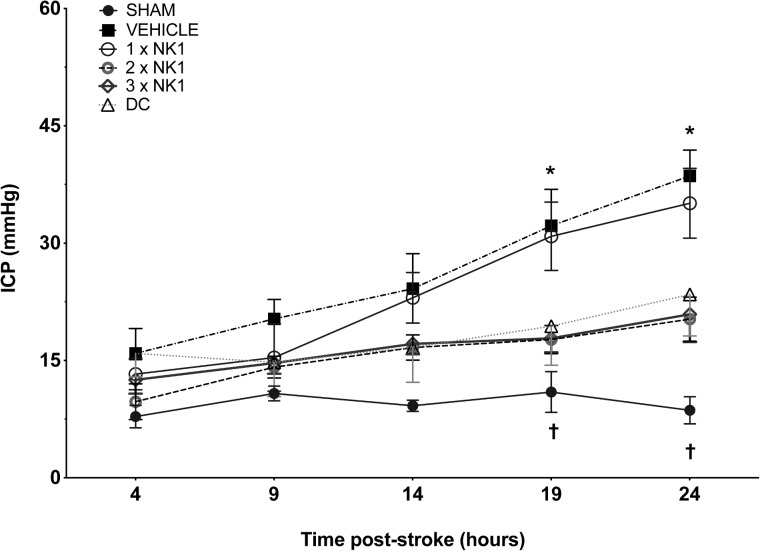
24 h ICP following MCAO. There was no significant difference in sham ICP across any of the time points. At 14 h post-stoke ICP was elevated in vehicle animals compared to sham (*p* < 0.05) and by 19 h post-stroke, ICP was significantly elevated in vehicle (*p* < 0.001) and 1×NK1 (*p* < 0.01) treated animals when compared to 2×NK1 (*p* < 0.05), 3×NK1 (*p* < 0.05) and DC treatment groups (*p* < 0.05). By 24 h, ICP remained significantly elevated in vehicle animals compared to sham (*p* < 0.0001), 2×NK1 (*p* < 0.01), 3×NK1 (*p* < 0.01) and DC (*p* < 0.05) treatment groups. ICP also remained elevated at 24 h post-stroke in 1×NK1 treatment groups compared to sham (*p* < 0.001) and 2×NK1 (*p* < 0.05). Data presented as mean ± SD. ^∗^*p* < 0.05 compared to sham; ^†^*p* < 0.05 compared to vehicle.

Intracranial pressure remained physiologically stable across all time points in sham animals (9 ± 3 mmHg). At both 4 and 9 h post-stroke, there was no significant difference in ICP between sham or any of the stroke groups. By 14 h following injury, ICP in vehicle animals was significantly elevated (24 ± 11 mmHg) compared to shams (10 ± 3 mmHg) (*p* = 0.019). However, no difference in ICP was seen between vehicle or any of the treatment groups at this time-point (Figure 1).

Intracranial pressure in vehicle animals continued to rise and by 19 h post-stroke measured 32 ± 11 mmHg, which was significantly elevated (*p* = 0.0002) compared to shams recording an ICP of 13 ± 6 mmHg. By 19 h there was a significant reduction in ICP seen in 2×NK1 (18 ± 4 mmHg; *p* = 0.013), 3×NK1 (17 ± 7 mmHg; *p* = 0.015), and DC (19 ± 11 mmHg; *p* = 0.042) treated animals compared to vehicle. Conversely, there was no reduction (*p* > 0.05) in ICP seen following 1×NK1 treatment (31 ± 9 mmHg) compared to vehicle, with ICP remaining significantly elevated compared to sham (*p* = 0.002).

By 24 h post-stroke, ICP in vehicle animals had continued to rise (39 ± 7 mmHg) and remained significantly elevated compared to shams (12 ± 8 mmHg; *p* < 0.001), DC (23 ± 12 mmHg; *p* = 0.013), 2×NK1 (20 ± 6 mmHg; *p* < 0.001), and 3×NK1 (19 ± 7 mmHg; *p* < 0.005) treated animals. ICP continued to rise in the 1×NK1 treatment group (35 ± 9 mmHg) and remained significantly elevated compared to both shams (*p* < 0.001) and 2×NK1 (*p* = 0.029) treatment groups. ICP in 2×NK1 and 3×NK1 treatment groups was comparable (*p* > 0.05) to DC across all time points following stroke. No rebound increases in ICP were observed following 2×NK1 or 3×NK1 treatment at any time-point post-stroke.

### MRI

**Figure 2:**
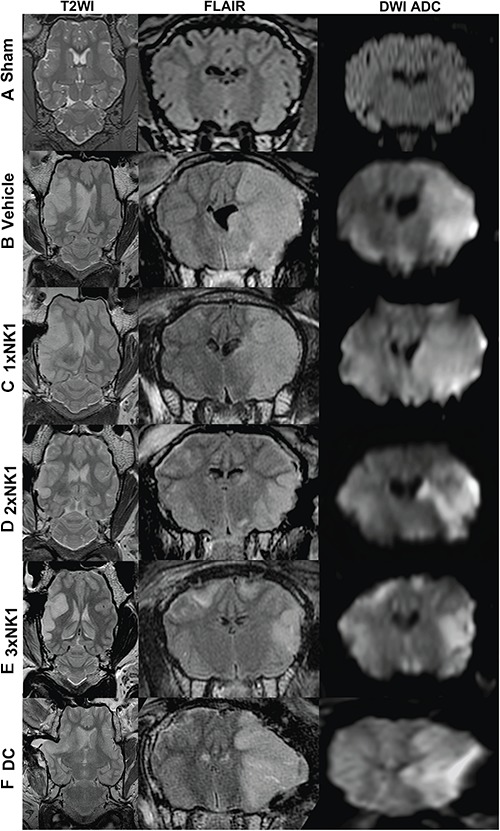
MRI findings 24 h post-MCAO. There was no evidence of hyperintensity or tissue deformation seen in sham animals **(A)**. Midline shift was evident in all stroke animals as seen on axial T2-weighted images **(B–D)**. Cerebral edema as shown in coronal FLAIR MRI images was greater in vehicle **(B)**, DC **(F)** and 1×NK1 **(C)** groups compared with 2×NK1 **(D)** and 3×NK1 **(E)** groups. This was associated with a smaller diffusion lesion volume as shown in DWI ADC images in the coronal plane. Note the transcalvarial herniation through the craniotomy site in DC treated animals **(F)**.

Magnetic resonance imaging was unremarkable in sham animals (*n* = 4) with no evidence of cerebral edema, midline shift or infarction (Figure 2A). All stroke animals showed evidence of hyperintensity on T2-weighted and FLAIR images in the territory MCA, indicative of the infarct core and surrounding vasogenic edema (Figure 2). In addition, DC treated animals showed evidence of transcalvarial herniation through the craniectomy site (Figure 2D). No animals showed evidence of tonsillar herniation or brain stem compression on MRI (data not shown).

**Figure 3:**
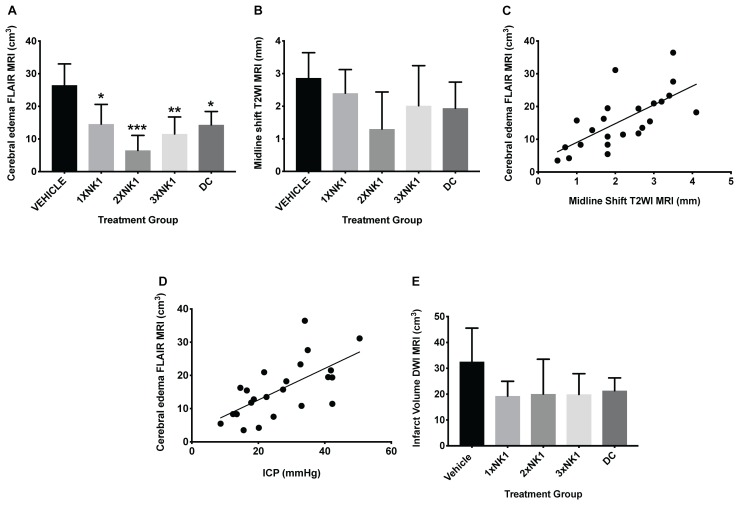
Cerebral edema and infarct volume findings at 24 h post-MCAO. Cerebral edema volume, as measured on FLAIR MRI **(A)**, was decreased in 1×NK1 (*p* < 0.05), 2×NK1 (*p* < 0.001), 3×NK1 (*p* < 0.01), and DC (*p* < 0.05) treatment groups when compared to vehicle. There was no difference in midline shift in any of the vehicle or treatment groups post-stroke **(B)**. Despite this, there was a relative moderate positive correlation between cerebral edema and midline shift **(C)**. There was also a moderate positive correlation between cerebral edema and ICP measured at 24 h post-stroke **(D)**. There was no significant difference in infarct volume **(E)** as measured on DWI MRI between any treatment groups. ^∗^*p* < 0.05, ^∗∗^*p* < 0.01 and ^∗∗∗^*p* < 0.001 compared to vehicle.

Cerebral edema calculated on FLAIR MRI (Figure 3 and Table 2) was reduced in DC (*p* = 0.012) and 1×NK1 treatment groups (*p* = 0.042) when compared to vehicle (Figure 3A). However, treatment with 2×NK1 (*p* = 0.001) and 3×NK1 (*p* = 0.001) was associated with a more significant reduction in cerebral edema volume compared to vehicle (Figure 3A). Midline shift was evident in all stroke animals (Table 2) with no statistical significance seen between groups (*p* = 0.2; Figure 3B). Nevertheless, there was a moderate positive relationship between the volume of cerebral edema and the degree of midline shift (*r* = 0.46; Figure 3C). No significant differences (*p* = 0.17) were observed in infarct volume across any of the treatment groups following stroke, as measured on DWI MRI (Figure 3D).

**Table 2:** MRI characteristics post-MCAO.

Treatment	Edema FLAIR MRI (cm^3^)	Midline Shift T2 (mm)	Infarct DWI MRI (cm^3^)
**Vehicle**			
86	19.48	1.8	33.9
87	36.45	3.5	24.5
60	20.96	3	25.9
66	31.14	2	34.4
57	27.61	3.5	20.1
59	23.32	3.4	56.5
Mean ± SD	25.56 ± 6.10	2.87 ± 0.71	32.55 ± 11.85
**1×NK1**			
69	10.84	1.8	22.1
75	21.51	3.2	23
88	11.44	2.2	12.7
Mean ± SD	14.60 ± 6.0	2.40 ± 0.72	19.27 ± 5.70
**2×NK1**			
70	4.230	0.8	8.3
72	3.520	0.5	17.2
91	11.789	2.6	34.7
Mean ± SD	6.51 ± 4.58	1.30 ± 1.14	20.07 ± 13.43
**3×NK1**			
71	5.49	1.8	11.6
73	13.50	2.7	20.7
74	7.56	0.7	10.6
76	8.33	1.1	20.8
89	18.23	4.1	32.1
90	16.27	1.7	23.7
Mean ± SD	11.56 ± 5.17	2.02 ± 1.23	19.92 ± 8.0
**DC**			
101	19.38	2.6	26.8
102	15.46	2.9	23.7
105	15.74	1	16.6
106	12.80	1.4	15.6
107	8.36	1.8	24
Mean ± SD	14.35 ± 4.08	1.94 ± 0.78	21.34 ± 4.95

### Histological Analysis

#### H&E

**Figure 4:**
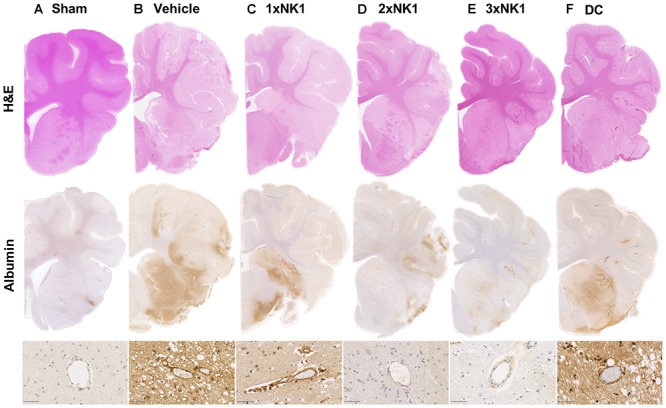
Hematoxylin and Eosin staining and albumin immunoreactivity in **(A)** sham, **(B)** vehicle, **(C)** 1×NK1, **(D)** 2×NK1, **(E)** 3×NK1, and **(F)** DC animals. H&E staining shows evidence of pallor and reduced gray white matter differentiation, which is especially prominent in vehicles and 1×NK1 treated animals. Enhanced albumin extravasation was seen macroscopically in vehicle, 1×NK1 and DC treated animals compared to sham, 2×NK1 and 3×NK1 treatment groups. This pattern of albumin staining is consistent with the perivascular staining seen in microscopic images. Scale bar 50 μm, 40× magnification.

There was no evidence of tissue injury or infarction in sham tissue (Figure 4A). Following stroke (Figure 4B) there was marked tissue pallor and loss of gray-white matter differentiation, indicative of infarction within the MCA territory of the right hemisphere. 1×NK1 (Figure 4C) treatment tissue was comparable to vehicles, however, there was an increased preservation of tissue, in particular the sub-cortical white matter, in the 2×NK1 (Figure 4D), 3×NK1 (Figure 4E) and DC (Figure 4F) treated groups.

#### Albumin

There was minimal evidence of albumin extravasation in sham tissue (Figure 4A). Following stroke, marked albumin extravasation, indicative of BBB breakdown and subsequent vasogenic cerebral edema formation, was observed both macroscopically within the infarcted territory and microscopically in the perivascular tissue of the infarct in vehicles (Figure 4B), 1×NK1 (Figure 4C) and DC (Figure 4F), when compared to shams (Figure 4A), 2×NK1 (Figure 4D) and 3×NK1 (Figure 4E) treatment groups.

#### SP

**Figure 5:**
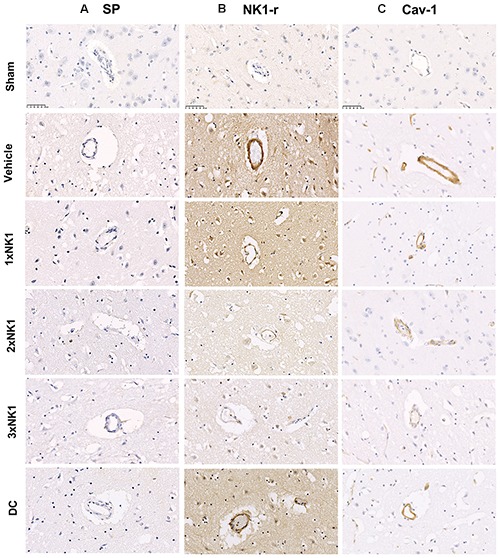
SP, NK1-r and caveolin-1 immunoreactivity. There was no identifiable increase in SP, NK1-r or cav-1 immunoreactivity in sham animals. Low levels of SP immunoreactivity were seen perivascularly in vehicle, 1×NK1, 2×NK1, 3×NK1 and DC groups **(A)**. This was concordant with an increase in NK1-r immunoreactivity, which was most prominent in vehicle, 1×NK1 and DC animals **(B)**. Cav-1 immunoreactivity was significantly enhanced in 1×NK1, 2×NK1 and DC treatment groups when compared to vehicle and 1×NK1 **(C)**. Scale bar: 50 μm, 40× magnification.

There was low to no observable immunoreactivity of SP in sham tissue (Figure 5A). Furthermore, similarly low levels of SP immunoreactivity were observed in the infarcted hemisphere of vehicle and each of the treatment groups following stroke.

#### NK1-r

Low levels of perivascular NK1-r immunoreactivity were observed in sham tissue (Figure 5B). Following stroke, an increase in perivascular NK1-r immunoreactivity was observed in vehicle and DC treated groups, which was not observed in any of the NK1 treatment groups.

#### Cav-1

Little to no perivascular cav-1 immunoreactivity was observed in sham tissue (Figure 5C). Following stroke, a marked increase in perivascular cav-1 immunoreactivity was observed in vehicles (E), 1×NK1 (F) and DC (L) tissue when compared to sham (D), 2×NK1 (J) and 3×NK1(K) tissue.

## Discussion

In this study we have demonstrated that administration of two or three boluses of an NK1-r antagonist is effective in reducing ICP following stroke in a clinically relevant ovine model. This reduction in ICP following treatment was associated with improved BBB integrity, as shown by albumin immunohistochemistry, and a trend toward reduction in the volume of cerebral edema in the infarcted hemisphere. These findings corroborate previous experimental (Vink et al., 2003; Nimmo et al., 2004; Turner et al., 2006, 2011; Donkin et al., 2009; Corrigan et al., 2012, 2016b; Turner and Vink, 2012, 2014) and clinical (Lorente et al., 2015, 2016) studies to further support the role of SP in the pathogenesis of elevated ICP, cerebral edema and increased permeability of the BBB following acute CNS injury. Indeed, the reduction in ICP following treatment observed in this study is consistent with findings in an ovine model of TBI (Vink et al., 2017). Taken together, we have now successfully demonstrated the efficacy of NK1-r antagonist treatment for the reduction of cerebral edema and its consequences in both rodent and sheep models of stroke (Turner et al., 2006, 2011; Turner and Vink, 2012, 2014) and TBI (Donkin et al., 2009; Corrigan et al., 2012, 2016b; Vink et al., 2017). Taken together these findings clearly demonstrate that SP release is a ubiquitous feature of acute CNS injury and that blockade of this pathway is able to preserve BBB integrity and halt the development of cerebral edema and subsequent rise in ICP.

Despite the clear treatment effect of blocking the NK1-r to reduce ICP in this study, we did not observe a significant increase in perivascular SP immunoreactivity within the infarcted territory in any of the treatment groups following stroke. This is in keeping with our previous findings in a rodent model of MCAO (Turner, 2007), where minimal SP immunoreactivity was observed following permanent MCAO, yet profound perivascular staining was identified following transient MCAO with reperfusion. These observations are most likely due to the fact that in the setting of permanent MCAO, the release and peak in SP occurs acutely due to the severity of the ischemic insult. As such, SP has likely been degraded by the time of tissue harvest at 24 h post-stroke. In comparison, in previous rodent transient MCAO studies, perivascular SP immunoreactivity was observed primarily in the penumbral tissue. The relative proportion of penumbra to ischemic core is likely to be much less following permanent compared to transient MCAO due to the extensive collateral failure with longer durations of ischemia. Not surprisingly, this suggests that the magnitude and timing of SP release in the neurogenic inflammatory response is dependent upon the duration and severity of the ischemic insult (Corrigan et al., 2016b). Indeed, following vascular occlusion, temporary changes in transient receptor potential channels of c-fibers as a result of alterations in temperature, pH and ligand binding instigates release of SP (Hokfelt et al., 1975). When there is no reperfusion, compromised cells undergo ischemic necrosis quickly with associated acute changes in cellular function, leading to a rapid return of receptor channel potential and decrease in SP release. Comparatively, in the setting of transient MCAO, compromised cells in the penumbra experience long durations of electrical silence and alterations in neuronal function, leading to sustained activation of c-fibers and persistent SP release (Astrup et al., 1981; Turner, 2007; Hofmeijer and Van Putten, 2012). Taken together, these findings may provide an explanation as to why SP was not observed on IHC in this study.

We did not observe any effect on infarct volume following NK1-r antagonist treatment, irrespective of the number of boluses administered. This is not surprising considering that we used a permanent stroke model and given that the NK1-r antagonist is targeting BBB disruption and not other pathways in the ischemic injury cascade. Indeed, a transient stroke model with reperfusion followed by delivery of an NK1-r antagonist may yield different results on infarct volume. Nevertheless, we also did not observe an effect on infarct volume at 24 hrs following 2 h MCAO thread occlusion in our rodent studies (Turner et al., 2011). In these previous rat studies, however, we did demonstrate that increased perivascular SP immunoreactivity was associated with increased BBB permeability, profound cerebral edema and persistent functional deficits (Turner et al., 2006, 2011; Turner and Vink, 2012, 2014).

In this study we have identified that increased BBB permeability was sustained to 24 h post-stroke in vehicle-treated animals, as identified by the increased albumin extravasation. Furthermore, the corresponding increase in perivascular caveolin-1 immunoreactivity in the vehicle group may provide an explanation for persistent changes barrier permeability seen. Caveolae are invaginations of the plasma membrane present in endothelial cells, including those that comprise the BBB, with a key role in regulating transcytosis of large molecules, including albumin, across the barrier (Abbott et al., 2006). Cav-1 is an integral protein for caveolae formation, with upregulation associated with enhanced albumin extravasation and the development of vasogenic cerebral edema (Nag et al., 2007). Furthermore, cav-1 is shown to be upregulated following rodent cortical-cold injury and feline TBI, despite maintenance of tight junction (TJ) integrity (Povlishock et al., 1978; Nag et al., 2007), suggesting that physical breakdown of the barrier through loss of TJ is not necessary for the development of cerebral edema. The NK1-r is located within caveolae, suggesting that its activation may play a role in regulating transcytosis (Monastyrskaya et al., 2005; Kubale et al., 2007). Indeed, this is consistent with the observation of reduced barrier permeability following NK1-r antagonist administration (Donkin et al., 2007; Turner et al., 2011; Turner and Vink, 2012, 2014; Corrigan et al., 2016a; Vink et al., 2017). It is proposed that SP release following acute CNS injury leads to NK1-r activation, including those located within the caveolae of endothelial cells. This precipitates enhanced transcytosis of albumin across the barrier, altering the osmotic gradient leading to the development of vasogenic edema. Inhibition of transcytosis-mediated albumin extravasation through administration of the NK1-r antagonist attenuates unfavorable alterations in osmotic gradient across the barrier, thus preventing the abnormal accumulation of water in the parenchyma and subsequent development of edema and rise in ICP.

As previously mentioned, in this study we were able to reliability measure changes in ICP throughout the 24 h monitoring period. Clinical studies investigating fluctuations in ICP following malignant MCA stroke have recorded pressures as high as 43 mmHg in patients that died, compared with 28 mmHg in survivors (Hacke et al., 1996). In the present study pressures as high as 50 mmHg were recorded in vehicle animals, however, two or three boluses of the NK1-r antagonist were sufficient to sustainably reduce ICP to less than 30 mmHg. Furthermore, 2×NK1 treated animals recorded pressures of less than 20 mmHg throughout the 24 h monitoring period, with the exception of one animal, and 3 boluses of the antagonist was sufficient to maintain pressures below 25 mmHg, again with the exception of one animal who recorded a maximum pressure of 28 mmHg. One dose of the NK1-r antagonist, however, was insufficient to produce a treatment effect with pressures averaging 35 mmHg. These findings suggest that multiple boluses of the NK1-r antagonist is a potentially viable therapeutic strategy to reduce elevated ICP following stroke, with the ability to bring about a clinically meaningful and sustainable reduction in pressure comparable to that observed in surviving patients (Hacke et al., 1996).

Furthermore, the reduction in ICP we observed with repeated NK1-r boluses was comparable to surgical decompression, which is encouraging given the limitations of current available pharmacotherapies used for ICP management, often necessitating early DC. Indeed, DC performed before clinical signs of herniation is shown to improve functional outcomes however surgery performed after onset of clinical deterioration may be too late to yield beneficial outcomes (Shah et al., 2019). Furthermore, although DC is the most powerful tool currently available to combat elevated ICP, the procedure is highly invasive, benefits remain controversial and the long-term implications of the procedure on ICP dynamics are not well understood (Funchal et al., 2018; Lilja-Cyron et al., 2019; Shah et al., 2019). A pooled analysis of the randomized control trials DECIMAL, HAMLET, and DESTINY, compared early DC with best available conventional medical management in patients with evidence of MCA territory hypersensitivity and a National Institute of Health Stroke Scale (NIHSS) > 15/20 (Jüttler et al., 2007; Vahedi et al., 2007a,b; Hofmeijer et al., 2009; Geurts et al., 2013). These studies found that that DC significantly reduced fatality rates and improved functional outcome compared with conventional pharmacotherapy alone. However, whilst some studies report reduced mortality and improved survival, it is at the cost of a higher number of individuals that are moderately severely disabled following the procedure (Vahedi et al., 2007a; Kurland et al., 2015). This becomes an issue of increasing concern given that DC is associated with higher mortality rates in those aged greater than 60 years of age, the most prevalent stroke patient population (Hofmeijer et al., 2009; Benjamin et al., 2018). The correlation between age and functional outcome remains an extremely important pre-treatment prognostic factor in deciding if patients should undergo DC (Chen et al., 2007), further highlighting the need for development of new therapies that can be administered to a wider patient population in a safe and timely manner, eliminating the need for surgical decompression and risk of associated morbidity. In this study we have demonstrated that repeated dosing of NK1-r antagonist treatment was able to reduce ICP comparable to DC surgery, thus providing an alternate treatment strategy, circumventing the need for invasive surgery.

### Limitations

Although we have established that the NK1-r antagonist is an effective strategy to lower ICP and we have previously determined that the ovine model is viable candidate for the screening of promising novel therapeutics, we must acknowledge the limitations of the study.

Due to the invasive nature of the surgery and significant deficits that ensued following permanent MCAO, animals were required to be maintained under anesthesia for the duration of the experiment. We recognize that human patients are rarely under the influence of anesthesia on stroke onset, and that the use of anesthetic agents may affect outcome measures. Despite this, the agents used for induction and maintenance were chosen to reduce any potential neuroprotective or damaging effects, as previously described in detail (Wells et al., 2015). Indeed, as ketamine is known for its neuroprotective properties, and inhalation isoflurane for its sub-neuroprotection and association with reduced blood pressure, the combination was used to bring about a countering effect. The synergistic use of these agents permitted adequate anesthesia with controlled MABP whilst preventing inadvertent neuroprotection as a confounding factor, an important consideration when modeling acute ischemic stroke. It must be acknowledged, however, that we did observe significant differences in MABP across several of the groups. Nevertheless, these alterations in MABP did not follow the same pattern as the alterations in ICP so it is therefore unlikely that they significantly contributed to the observed treatment effects. The differences in MABP observed between groups most likely reflects both the within group and between group heterogeneity in the amount of isoflurane required to maintain twilight anesthesia during surgery whilst balancing with ketamine to maintain adequate blood pressure.

Furthermore, only female animals were used in the present study due to the inherent need for catheterization of animals during long-duration anesthesia. Females were selected preferentially over males upon veterinary advice given the difficulty in catheterizing the highly convoluted male urethra.

Though we were able to establish a significant increase in ICP following ovine stroke, MRI findings were not strongly correlated with ICP. Although the relationship between ICP and cerebral edema well established and promising in this study, we acknowledge that the small sample sizes in this study may have led to a low statistical power. Furthermore, it must be noted that this is frequently seen in clinical stroke cases, where MRI and computed tomography (CT) findings are not always a good predictor of ICP. Due to facility access, MRI was not possible in the entire cohort, leading to uneven group sizes. Despite a larger cohort available for ICP in both treatment and vehicle groups, only animals with matching ICP/MRI data were included, leading to variations in group sizes. Furthermore, the reliable measurement of cerebral edema on MRI at a single time-point is somewhat contentious, as the hyperintensity of vasogenic edema is difficult to distinguish from the lesion itself. The measurement of the entire FLAIR lesion volume therefore includes areas of infarction as well as edema, and is thus an indirect measure of edema volume. A more robust approach to measuring edema evolution would be to perform sequential MRI’s and evaluate the change in diffusion lesion volume between 2 times points following reperfusion, which was not possible in this study due to cost. It should also be acknowledged that whilst MRI findings did not correlate with ICP, we were able to observe MRI features similar to that seen clinically, including transcalvarial herniation in DC treated animals and reduced midline shift. Finally, testing of multiple NK1-r antagonists was not feasible in this study given the time and cost involved in conducting sheep experiments. However, in our rodent studies of TBI we have previously tested 2 different NK1-r antagonists and recorded comparable results (Donkin, 2006) which provided the basis for the use of the agent in the present study.

For future studies we will work alongside the animal ethics committee of the South Australian Health and Medical Research Institute (SAHMRI) to develop a survival model of transient MCAO which obviates the need for long duration anesthesia, allowing for the study of conscious animals following induction of stroke and assessment of long-term functional changes.

## Conclusion

Multiple boluses of an NK1-r antagonist is effective in lowering ICP following ovine stroke, producing a reduction in ICP that is comparable to decompressive surgery. We propose that the mechanism by which the NK1-r antagonist is exerting its effect is largely via caveolae mediated albumin transcytosis. Administration of the NK1-r is thus preventing abnormal albumin extravasation from the vasculature to the brain parenchyma and ameliorating the subsequent development of vasogenic edema and rise in ICP. These findings suggest that NK1-r antagonist treatment may represent a novel intervention for the management of elevated ICP following stroke.

## Data Availability

All datasets generated for this study are included in the manuscript and/or the supplementary files.

## Ethics Statement

All experimental procedures were approved by the Animal Ethics Committees of the University of Adelaide (M-2011-240) and the South Australian Health and Medical Research Institute (SAHMRI; SAM104/11) and conducted according to guidelines established for the use of animals in experimental research as outlined by the Australian National Health and Medical Research Council code of practice for the care and use of animals for scientific purposes (8th edition, 2013).

## Author Contributions

AW, RV, RT, and AL conceived and designed the experiments. AW, RT, and AL carried out the experiments. AS-A, NY, JH, and RT analyzed the data. AS-A, RT, and AL wrote the manuscript.

## Conflict of Interest Statement

The authors declare that the research was conducted in the absence of any commercial or financial relationships that could be construed as a potential conflict of interest.

## References

[B1] Abbott N. J. Ronnback L. Hansson E. (2006). Astrocyte-endothelial interactions at the blood-brain barrier. *Nat. Rev. Neurosci.* 7 41–53. 10.1038/nrn1824 16371949

[B2] Alves R. V. Campos M. M. Santos A. R. Calixto J. B. (1999). Receptor subtypes involved in tachykinin-mediated edema formation. *Peptides* 20 921–927. 10.1016/s0196-9781(99)00082-0 10503769

[B3] Astrup J. Siesjo B. K. Symon L. (1981). Thresholds in cerebral ischemia - the ischemic penumbra. *Stroke* 12 723–725. 10.1161/01.str.12.6.723 6272455

[B4] Bardutzky J. Schwab S. (2007). Antiedema therapy in ischemic stroke. *Stroke* 38 3084–3094. 10.1161/strokeaha.107.490193 17901384

[B5] Benjamin E. J. Virani S. S. Callaway C. W. Chamberlain A. M. Chang A. R. Cheng S. (2018). Heart disease and stroke statistics-2018 update: a report From the American heart association. *Circulation* 137 e67-e492. 10.1161/CIR.0000000000000558 29386200

[B6] Brogan M. Manno E. (2015). Treatment of malignant brain edema and increased intracranial pressure after stroke. *Cur. Treat. Options Neurol.* 17 1–11. 10.1007/s11940-014-0327-0 25398467

[B7] Chen C.-C. Cho D.-Y. Tsai S.-C. (2007). Outcome of and prognostic factors for decompressive hemicraniectomy in malignant middle cerebral artery infarction. *J. Clin. Neurosci.* 14 317–321. 10.1016/j.jocn.2005.05.024 17275311

[B8] Corrigan F. Leonard A. Ghabriel M. Van Den Heuvel C. Vink R. (2012). A substance P antagonist improves outcome in female sprague dawley rats following diffuse traumatic brain injury. *CNS Neurosci. Ther.* 18 513–515. 10.1111/j.1755-5949.2012.00332.x 22672307 PMC6493367

[B9] Corrigan F. Mander K. A. Leonard A. V. Vink R. (2016a). Neurogenic inflammation after traumatic brain injury and its potentiation of classical inflammation. *J. Neuroinflammation* 13:264. 27724914 10.1186/s12974-016-0738-9 PMC5057243

[B10] Corrigan F. Vink R. Turner R. J. (2016b). Inflammation in acute CNS injury: a focus on the role of substance P. *Br. J. Pharmacol.* 173 703–715. 10.1111/bph.13155 25827155 PMC4742293

[B11] Das S. Mitchell P. Ross N. Whitfield P. C. (2019). Decompressive hemicraniectomy in the treatment of malignant middle cerebral artery infarction: a meta-analysis. *World Neurosurgery* 123 8–16. 10.1016/j.wneu.2018.11.176 30500591

[B12] Donkin J. D. (2006). *The effects of the neuropeptide substance P on outcome following experimental traumatic brain injury in rats.* Adelaide: University of Adelaide.

[B13] Donkin J. J. Nimmo A. J. Cernak I. Blumbergs P. C. Vink R. (2009). Substance P is associated with the development of brain edema and functional deficits after traumatic brain injury. *J. Cereb. Blood Flow Metab.* 29 1388–1398. 10.1038/jcbfm.2009.63 19436311

[B14] Donkin J. J. Turner R. J. Hassan I. Vink R. (2007). Substance P in traumatic brain injury. *Prog. Brain Res.* 161 97–109. 17618972 10.1016/S0079-6123(06)61007-8

[B15] Dostovic Z. Dostovic E. Smajlovic D. Ibrahimagic O. C. Avdic L. (2016). Brain edema after ischaemic stroke. *Med. Arch.* 70 339–341. 27994292 10.5455/medarh.2016.70.339-341 PMC5136437

[B16] Funchal B. F. Alves M. M. Suriano I. C. Chaddad-Neto F. E. Ferraz M. Silva G. S. (2018). Intracranial pressure following decompressive hemicraniectomy for malignant cerebral infarction: clinical and treatment correlations. *Arq Neuropsiquiatr.* 76 812–815. 10.1590/0004-282X20180132 30698203

[B17] Geurts M. Van Der Worp H. B. Kappelle L. J. Amelink G. J. Algra A. Hofmeijer J. (2013). Surgical decompression for space-occupying cerebral infarction: outcomes at 3 years in the randomized HAMLET trial. *Stroke* 44 2506–2508. 10.1161/STROKEAHA.113.002014 23868265

[B18] Hacke W. Schwab S. Horn M. Spranger M. De Georgia M. Von Kummer R. (1996). ‘Malignant’ middle cerebral artery territory infarction: clinical course and prognostic signs. *Arch. Neurol.* 53 309–315. 8929152 10.1001/archneur.1996.00550040037012

[B19] Hofmeijer J. Kappelle L. J. Algra A. Amelink G. J. Van Gijn J. Van Der Worp H. B. (2009). Surgical decompression for space-occupying cerebral infarction (the hemicraniectomy after middle cerebral artery infarction with life-threatening edema trial [HAMLET]): a multicentre, open, randomised trial. *Lancet. Neurol.* 8 326–333. 10.1016/S1474-4422(09)70047-X 19269254

[B20] Hofmeijer J. Van Putten M. J. (2012). Ischemic cerebral damage: an appraisal of synaptic failure. *Stroke* 43 607–615. 10.1161/STROKEAHA.111.632943 22207505

[B21] Hokfelt T. Broberger C. Xu Z. Q. Sergeyev V. Ubink R. Diez M. (2000). Neuropeptides–an overview. *Neuropharmacology* 39 1337–1356. 10.1016/s0028-3908(00)00010-1 10818251

[B22] Hokfelt T. Kellerth J. O. Nilsson G. Pernow B. (1975). Substance P: localisation in the central nervous system and in some primary sensory neurones. *Science* 190 889–890. 10.1126/science.242075 242075

[B23] Hudetz J. A. Pagel P. S. (2010). Neuroprotection by ketamine: a review of the experimental and clinical evidence. *J. Cardiothorac. Vasc. Anesth.* 24 131–142. 10.1053/j.jvca.2009.05.008 19640746

[B24] Jüttler E. Schwab S. Schmiedek P. Unterberg A. Hennerici M. Woitzik J. (2007). Decompressive surgery for the treatment of malignant infarction of the middle cerebral artery (DESTINY): a randomized, controlled trial. *Stroke* 38 2518–2525. 10.1161/strokeaha.107.485649 17690310

[B25] Kubale V. Abramovic Z. Pogacnik A. Heding A. Sentjurc M. Vrecl M. (2007). Evidence for a role of caveolin-1 in neurokinin-1 receptor plasma-membrane localization, efficient signaling, and interaction with beta-arrestin 2. *Cell Tissue Res.* 330 231–245. 10.1007/s00441-007-0462-y 17713785

[B26] Kurland D. B. Khaladj-Ghom A. Stokum J. A. Carusillo B. Karimy J. K. Gerzanich V. (2015). Complications associated with decompressive craniectomy: a systematic review. *Neurocrit. care* 23 292–304. 10.1007/s12028-015-0144-7 26032808 PMC4704457

[B27] Lewis K. M. Turner R. J. Vink R. (2013). Blocking neurogenic inflammation for the treatment of acute disorders of the central nervous system. *Int. J. Inflam.* 2013:578480. 10.1155/2013/578480 PMC3681302 23819099

[B28] Lilja-Cyron A. Andresen M. Kelsen J. Andreasen T. H. Fugleholm K. Juhler M. (2019). Long-term effect of decompressive craniectomy on intracranial pressure and possible implications for intracranial fluid movements. *Neurosurgery* 10.1093/neuros/nyz049 [Epub ahead of print]. 30768137

[B29] Lorente L. Martín M. M. Almeida T. Hernández M. Ramos L. Argueso M. (2015). Serum substance p levels are associated with severity and mortality in patients with severe traumatic brain injury. *Crit. Care* 19:192. 10.1186/s13054-015-0911-z 25928056 PMC4424826

[B30] Lorente L. Martín M. M. Almeida T. Pérez-Cejas A. Ramos L. Argueso M. (2016). Serum Levels of Substance P and Mortality in Patients with a Severe Acute Ischemic Stroke. *Int.J. Mol. Sci.* 17:991. 10.3390/ijms17060991 27338372 PMC4926519

[B31] Matthews J. N. Altman D. G. Campbell M. J. Royston P. (1990). Analysis of serial measurements in medical research. *BMJ* 300 230–235. 10.1136/bmj.300.6719.230 2106931 PMC1662068

[B32] Monastyrskaya K. Hostettler A. Buergi S. Draeger A. (2005). The NK1 receptor localizes to the plasma membrane microdomains, and its activation is dependent on lipid raft integrity. *J. Biol. Chem.* 280 7135–7146. 10.1074/jbc.m405806200 15590676

[B33] Nag S. Venugopalan R. Stewart D. J. (2007). Increased caveolin-1 expression precedes decreased expression of occludin and claudin-5 during blood-brain barrier breakdown. *Acta Neuropathol.* 114 459–469. 10.1007/s00401-007-0274-x 17687559

[B34] Nimmo A. J. Cernak I. Heath D. L. Hu X. Bennett C. J. Vink R. (2004). Neurogenic inflammation is associated with development of edema and functional deficits following traumatic brain injury in rats. *Neuropeptides* 38 40–47. 10.1016/j.npep.2003.12.003 15003715

[B35] Povlishock J. T. Becker D. P. Sullivan H. G. Miller J. D. (1978). Vascular permeability alterations to horseradish peroxidase in experimental brain injury. *Brain Res.* 153 223–239. 10.1016/0006-8993(78)90404-3 687980

[B36] Schifilliti D. Grasso G. Conti A. Fodale V. (2010). Anaesthetic-related neuroprotection intravenous or inhalational agents? *CNS Drugs* 24 893–907. 10.2165/11584760-000000000-00000 20932063

[B37] Shah A. Almenawer S. Hawryluk G. (2019). Timing of decompressive craniectomy for ischemic stroke and traumatic brain injury: a review. *Front. Neurol.* 10:11. 10.3389/fneur.2019.00011 30740085 PMC6355668

[B38] Simard J. M. Kent T. A. Chen M. Tarasov K. V. Gerzanich V. (2007). Brain oedema in focal ischaemia: molecular pathophysiology and theoretical implications. *Lancet. Neurol.* 6 258–268. 10.1016/s1474-4422(07)70055-8 17303532 PMC2725365

[B39] Sorby-Adams A. J. Marcoionni A. M. Dempsey E. R. Woenig J. A. Turner R. J. (2017). The role of neurogenic inflammation in blood-brain barrier disruption and development of cerebral oedema following acute central nervous system (cns) injury. *Int. J. Mol. Sci.* 18:E1788. 10.3390/ijms18081788 PMC5578176 28817088

[B40] Sorby-Adams A. J. Vink R. Turner R. J. (2018). Large animal models of stroke and traumatic brain injury as translational tools. *Am. J. physiol. Regul. Integr. comp. physiol.* 315 R165–R190. 10.1152/ajpregu.00163.2017 29537289

[B41] Stumm R. Culmsee C. Schafer M. K. Krieglstein J. Weihe E. (2001). Adaptive plasticity in tachykinin and tachykinin receptor expression after focal cerebral ischemia is differentially linked to gabaergic and glutamatergic cerebrocortical circuits and cerebrovenular endothelium. *J. Neurosci.* 21 798–811. 10.1523/jneurosci.21-03-00798.2001 11157066 PMC6762313

[B42] Turner R. E. J. (2007). *Characterising the role of substance P in acute ischaemic stroke.* Thesis. University of Adelaide. Adelaide.

[B43] Turner R. J. Blumbergs P. C. Sims N. R. Helps S. C. Rodgers K. M. Vink R. (2006). Increased substance P immunoreactivity and edema formation following reversible ischemic stroke. *Acta Neurochir Suppl.* 96 263–266. 10.1007/3-211-30714-1_56 16671467

[B44] Turner R. J. Helps S. C. Thornton E. Vink R. (2011). A substance P antagonist improves outcome when administered 4 h after onset of ischemic stroke. *Brain Res.* 1393 84–90. 10.1016/j.brainres.2011.03.066 21466790

[B45] Turner R. J. Vink R. (2012). Combined tissue plasminogen activator and an NK1 tachykinin receptor antagonist: an effective treatment for reperfusion injury following acute ischemic stroke in rats. *Neuroscience* 220 1–10. 10.1016/j.neuroscience.2012.06.047 22750240

[B46] Turner R. J. Vink R. (2014). NK1 tachykinin receptor treatment is superior to capsaicin pre-treatment in improving functional outcome following acute ischemic stroke. *Neuropeptides* 48 267–272. 10.1016/j.npep.2014.07.002 25151181

[B47] Vahedi K. Hofmeijer J. Juettler E. Vicaut E. George B. Algra A. (2007a). Early decompressive surgery in malignant infarction of the middle cerebral artery: a pooled analysis of three randomised controlled trials. *Lancet. Neurol.* 6 215–222. 10.1016/s1474-4422(07)70036-4 17303527

[B48] Vahedi K. Vicaut E. Mateo J. Kurtz A. Orabi M. Guichard J.-P. (2007b). Sequential-design, multicenter, randomized, controlled trial of early decompressive craniectomy in malignant middle cerebral artery infarction (DECIMAL Trial). *Stroke* 38 2506–2517. 10.1161/strokeaha.107.485235 17690311

[B49] Vink R. Gabrielian L. Thornton E. (2017). The Role of Substance P in Secondary Pathophysiology after Traumatic Brain Injury. *Front. Neurol.* 8:304. 10.3389/fneur.2017.00304 28701994 PMC5487380

[B50] Vink R. Young A. Bennett C. J. Hu X. Connor C. O. Cernak I. (2003). Neuropeptide release influences brain edema formation after diffuse traumatic brain injury. *Acta Neurochir. Suppl.* 86 257–260. 10.1007/978-3-7091-0651-8_55 14753447

[B51] Wells A. J. Vink R. Blumbergs P. C. Brophy B. P. Helps S. C. Knox S. J. (2012). A surgical model of permanent and transient middle cerebral artery stroke in the sheep. *PLoS One* 7:e42157. 10.1371/journal.pone.0042157 22848737 PMC3407087

[B52] Wells A. J. Vink R. Helps S. Knox S. J. Blumbergs P. C. Turner R. J. (2015). Elevated intracranial pressure and cerebral edema following permanent mca occlusion in an ovine model. *PLoS One* 10:e0130512. 10.1371/journal.pone.0130512 26121036 PMC4486455

[B53] Xiao-feng Y. Yu Y. Wei-Wei H. Gu L. Jin-Fang X. Xue-Qun Z. (2005). Is decompressive craniectomy for malignant middle cerebral artery infarction of any worth? *J. Zhejiang Univ. Sci. B* 6 644–649. 10.1631/jzus.2005.b0644 15973766 PMC1389798

[B54] Xu S. Y. Pan S. Y. (2013). The failure of animal models of neuroprotection in acute ischemic stroke to translate to clinical efficacy. *Med. sci. Monit. Basic Res.* 19 37–45. 23353570 10.12659/MSMBR.883750 PMC3638705

[B55] Yushkevich P. A. Piven J. Hazlett H. C. Smith R. G. Ho S. Gee J. C. (2006). User-guided 3D active contour segmentation of anatomical structures: significantly improved efficiency and reliability. *Neuroimage* 31 1116–1128. 10.1016/j.neuroimage.2006.01.015 16545965

[B56] Zhang W. Neal J. Lin L. Dai F. Hersey D. P. Mcdonagh D. L. (2018). Mannitol in critical care and surgery over 50+ years: a systematic review of randomized controlled trials and complications with meta-analysis. *J. Neurosurg. Anesthesiol.* 31 273–284. 10.1097/ANA.0000000000000520 29952815

